# Cocaine Dependent Individuals and Gamblers Present Different Associative Learning Anomalies in Feedback-Driven Decision Making: A Behavioral and ERP Study

**DOI:** 10.3389/fpsyg.2013.00122

**Published:** 2013-03-18

**Authors:** Ana Torres, Andrés Catena, Antonio Cándido, Antonio Maldonado, Alberto Megías, José C. Perales

**Affiliations:** ^1^Learning, Emotion and Decision Research Group, Mind, Brain and Behavior Research Center/Centro de Investigación Mente, Cerebro y Comportamiento, University of GranadaGranada, Spain

**Keywords:** addiction, cocaine, gambling, reversal learning, feedback-related negativity, decision-making

## Abstract

Several recent studies have demonstrated that addicts behave less flexibly than healthy controls in the probabilistic reversal learning task (*PRLT*), in which participants must gradually learn to choose between a probably rewarded option and an improbably rewarded one, on the basis of corrective feedback, and in which preferences must adjust to abrupt reward contingency changes (reversals). In the present study, pathological gamblers (PG) and cocaine dependent individuals (CDI) showed different learning curves in the PRLT. PG also showed a reduced electroencephalographic response to feedback (Feedback-Related Negativity, FRN) when compared to controls. CDI’s FRN was not significantly different either from PG or from healthy controls. Additionally, according to Standardized Low-Resolution Electromagnetic Tomography analysis, cortical activity in regions of interest (previously selected by virtue of their involvement in FRN generation in controls) strongly differed between CDI and PG. However, the nature of such anomalies varied within-groups across individuals. Cocaine use severity had a strong deleterious impact on the learning asymptote, whereas gambling intensity significantly increased reversal cost. These two effects have remained confounded in most previous studies, which can be hiding important associative learning differences between different populations of addicts.

## Introduction

Response-outcome association learning tasks have been widely used to explore the cognitive and biological underpinnings of neuropsychiatric disorders (e.g., Everitt et al., 2001; Clark et al., 2004; Redish et al., 2007). The *probabilistic reversal learning task* (*PRLT*; Swainson et al., 2000) is a dynamic decision-making test (Hastie and Dawes, 2009) in which participants must learn to choose between two response options, one frequently rewarded (and infrequently punished), and the other infrequently rewarded (and frequently punished). Payoffs are administered in the form of real or play money, or virtual points. Once preferences are stable, reward/punishment contingencies reverse, in such a way that the advantageous option becomes disadvantageous, and *vice versa*, and learners must retune their preferences in accordance with the new contingencies.

Addicted individuals (and patients from other psychopathological and neurological populations) have been observed to display abnormal performance patterns in the reversal learning task. Some types of patients are slower than normal to readjust their preferences after a reversal. This *increased reversal cost* has been interpreted as a sign of goal-disengaged, habit-driven, error-insensitive, or perseverative behavior (Clarke et al., 2005, 2008). In other cases, pre-reversal learning asymptote has been observed to be abnormally low (e.g., Fernández-Serrano et al., 2012), or abnormally high (e.g., Verdejo-García et al., 2010). Although in many studies pre-asymptotic and asymptotic effects have not been dissociated (see Tsuchida et al., 2010; Torres et al., submitted; for similar arguments), there is broad consensus that the sort of dynamic decision-making processes involved in reversal learning tasks is crucial to understand the neuropsychology of addictive disorders (Ersche et al., 2008; Camchong et al., 2011; Izquierdo and Jentsch, 2012; Leeman and Potenza, 2012; Lucantonio et al., 2012). As also shown in this work, reversal learning tasks tackle on the type of balanced feedback sensitivity and learning flexibility that are needed for adaptive decision making in real life.

In spite of that, abnormal PRLT performance patterns do not seem to fully generalize across addictions. Separate sources of evidence seem to show that heavy gambling is preferentially linked to the increase of reversal cost (de Ruiter et al., 2009), whereas cumulative toxicity of cocaine generates more unspecific performance deviations and, particularly, less accurate decision making once asymptotic learning has been reached, prior to contingency reversal (accompanied by working memory and planning dysfunction; Fernández-Serrano et al., 2012). In a recent review, Leeman and Potenza (2012) have integrated these independent pieces of evidence, and have concluded that increased reversal costs are more frequent and robust in pathological gamblers (PG) than in drug-dependent individuals (see also Ersche et al., 2008).

The present study focuses on the coincidences and divergences between gambling and cocaine addiction, with regard to the anomalies they generate in reversal learning performance. A sample of cocaine dependent individuals (CDI) was compared against one of pathological gamblers (PG), and a group of matched healthy controls (HC) in a reversal learning task, at the behavioral and the electroencephalographic levels. To our knowledge, only two studies have directly compared cocaine users against PG in a battery of personality and neuropsychological tests (Albein-Urios et al., 2012a; Torres et al., 2013). However, no studies have directly compared matched groups of patients with the two disorders, between them and against a group of HC, in the PRLT.

There are several reasons to jointly study PG and CDI samples, but also to draw conclusions with some caution. First, parallelisms between these two addictions have been known for a long time. Some studies have found behavioral similarities, high comorbidity rates, and a partially common neurobiological and genetic etiology (see Hall et al., 2000; Potenza, 2008). For example, prospective family studies have observed that the percentage of future gamblers among children of gamblers doubles the population baseline (8 versus 4%). And, in parallel, children of gamblers tend to show a preference for stimulant drugs, so that the proportion of future cocaine users among children of gamblers doubles the population baseline (10 versus 5%; Jacobs et al., 1989). Complementarily, in a sample of 298 treatment-seeking cocaine abusers, Steinberg et al. (1992) found a prevalence of pathological gambling approximately 10 times larger than the rate of gamblers found in community samples.

As noted by Albein-Urios et al. (2012a), “the two disorders have also notable similarities in terms of subjective effects, reinforcing schedules, and temporal patterns of consumption […]. In these respects, cocaine addiction is arguably more similar to pathological gambling than other forms of drug dependence.” Moreover, direct experimental evidence shows that a game of chance can serve as an alternative reinforcer to smoking cocaine (Vosburg et al., 2010).

Second, these similarities seem to indicate that a comparison between PG and CDI could be helpful to disentangle vulnerability and toxicity effects of cocaine use in group comparison studies. This argument is based on the assumption that the neurobehavioral anomalies observed in PG samples are equivalent to those found in CDI samples *minus* the neurotoxic effects of cocaine. Still, this rationale is problematic, as far as it assumes that gambling does not have a cumulative impact on brain function (an assumption that goes against current evidence; see Robinson and Berridge, 2003; van Holst et al., 2010). Mere between-groups comparisons do not strictly allow such a type of conclusions.

And third, although only prospective and longitudinal studies can strictly discriminate vulnerability from cocaine/gambling exposure factors, studies comparing samples of addicts against HC can be informative if they meet some criteria. On the one hand, although complete matching between samples is virtually unattainable, it is important to select samples carefully. They must be completely separated in terms of key addictive behaviors (gamblers do not use cocaine, cocaine users do not gamble, and controls neither gamble nor use cocaine), and matched in terms sociodemographic variables, intellectual functioning, and absence of any other psychiatric disorders. And, on the other hand, chronic exposure to cocaine/gambling must be estimated on an individual basis. In this type of studies, the degree of exposure can be measured only retrospectively, but there exist interview-based methods to approximate it. These methods allow for the estimation of exposure-dependent effects on neurobehavioral anomalies (Verdejo-García et al., 2005). Estimation of exposure-dependent effects can help us to identify *acquired* individual differences caused by the progressive course of the addictive processes, that is, by toxicity, neuroadaptation, or sensitization.

In this work, we also recorded feedback-evoked electroencephalographic activity during reversal learning. The analysis of this activity is valuable in several senses. Evoked-related potentials (ERP) are sometimes more sensitive to between-condition differences than behavioral measures (see, for example, Karayanidis et al., 2000; Hajcak et al., 2005b). Accordingly, convergent psychophysiological and behavioral evidence is more conclusive than behavioral results alone, especially when behavioral effects are subtle. Furthermore, in the present case, there are also evidence-driven hypotheses about the potential biological substrate of reversal learning anomalies, and the candidate ERP components that best reveal such anomalies. Our interest in the feedback-related negativity (FRN) and its potential relation with reversal costs, is grounded on previous experimental evidence (Chase et al., 2011; Bismark et al., 2012; Hampshire et al., 2012). Finally, our attempts to identify the most likely anatomical origins of addiction-related FRN anomalies can be useful to link such anomalies to the malfunctioning of specific circuits in the brain (Schoenbaum et al., 2006).

In summary, in the present work we analyze in detail some dynamic features of reversal learning performance in PG and CDI, matched in potentially confounding factors, and compared against non-addicts. Our main aim is threefold: (1) To check for the existence of anomalies in reversal learning in both types of addicts. On this regard, we expect reversal cost to be more evident in PG than in cocaine users. (2) To explore the roles of gambling and cocaine exposure on specific components of reversal learning (specifically, reversal costs and asymptotic learning levels). As measures of chronic and acute exposure, *severity* (the estimation of the lifetime total amount gambled, or the total quantity of cocaine consumed) and *intensity* (mean amount of drug consumed/money gambled per month) scores will be obtained for all participants in the clinical groups. On the basis of the abovementioned evidence, we expect learning anomalies in the CDI group to be attributable to cocaine dosage exposure (and thus to correlate with cocaine use severity). Whether or not reversal cost depends on gambling intensity or severity remains an open question. And (3), to analyze the electroencephalographical response to feedback in the three groups. Although we can foretell the presence of FRN anomalies in the clinical groups (and associated abnormal brain activations), whether or not such anomalies differ across the clinical groups also remains to be tested.

## Materials and Methods

### Participants and procedure

Cocaine dependent individuals (*n* = 20) were recruited from the *Proyecto Hombre* rehabilitation centers in Granada and Málaga (Spain) between January 2011 and December 2012. PG (*n* = 21) were recruited from *AGRAJER* (Granadian Association of Gamblers in Rehabilitation, Granada, Spain) between October 2010 and December 2012. Controls (*n* = 23) were recruited by incidental sampling, in such a way that their sociodemographic characteristics were not far from the clinical groups.

The inclusion criteria were (i) meeting DSM-IV criteria for cocaine dependence (CDI group) or pathological gambling (PG group) – as assessed by the Structured Clinical Interview for DSM-IV Disorders – Clinician Version (SCID; First et al., 1997); (ii) having a minimum abstinence interval of 15 days for all substances of abuse except nicotine, as determined by weekly urine toxicological tests (CDI) or cross validated therapist- and self-reports (PG). Exclusion criteria were: (i) the presence of any other Axis I or Axis II comorbid disorders with the exception of nicotine dependence; (ii) the presence of history of head injury or any diseases affecting the central nervous system. The study counted with explicit permission from the University of Granada’s Ethics Committee. Prior to psychological and neuropsychological assessment, all participants were informed about the objectives and characteristics of the study, and signed an informed consent form. All of them were compensated with 36€ for their participation, independently of performance.

In order to assess the degree of matching between-groups, participants were also assessed using the Kaufman Brief Intelligence Test (K-BIT), and were questioned about their age, and number of education years. Table 1 displays main descriptive data for all the relevant variables in the three groups. The three groups were matched on sociodemographic variables, but not on usage of other drugs. As shown in the table, the group differences in alcohol and cannabis use were globally significant, with CDI being the group with larger alcohol and cannabis consumption.

**Table 1:** **Sociodemographic, psychometric, and drug use differences between healthy controls, HC; pathological gamblers, PG; and cocaine dependent individuals, CDI**.

*Group*								
	***HC***		***PG***		***CDI***			
**SOCIODEMOGRAPHIC VARIABLES**								
*n*	23		21		20			
Proportion of females	0.09		0.10		0.00			
	**Mean**	**SD**	**Mean**	**SD**	**Mean**	**SD**	***F***	***p***
	
Age	30.13	8.63	31.43	5.92	34.75	6.51	2.31	0.11
Education years	14.55	3.16	13.90	4.66	15.05	4.21	0.42	0.66
**INTELLECTUAL PERFORMANCE**								
IQ (K-BIT)	106.25	10.22	101.10	9.07	105.35	9.39	1.77	0.18
**DRUG USE PATTERNS**								
Alcohol monthly use (ethanol units/month)	44.02	42.81	99.14	73.87	158.20	118.88	10.18	<0.01*
Cannabis monthly use (joints/month)	13.00	26.61	8.39	25.96	67.00	63.71	12.54	<0.01*
Addiction course duration (years)			7.79	5.51	10.48	5.04	2.65	0.11
Abstinence duration (months)			5.64	3.51	6.41	4.32	0.39	0.53

*Addiction and abstinence durations refer to the clinically significant addictive behavior (gambling for PG, and cocaine use for CDI). p-values in bold are statistically significant*.

The procedure went as follows: upon consent, participants were instructed about the general procedure, and then questioned about the abovementioned sociodemographic variables. The K-BIT, Interview for Research on Addictive Behavior (IRAB), and the IRAB-equivalent gambling-related questions were administered together, in a random order. A fourth psychometric instrument, the UPPS-P questionnaire on impulsive behavior, was administered intertwined with these, also in a random position. The PRLT and a second neuropsychological task (the Go/No-go motor inhibition task) were administered together. Half the participants performed the neuropsychological tasks first, in a random order, followed by the psychometric instruments. The other half were assessed with the psychometric tools first, and then performed the two neuropsychological tasks.

The UPPS-P and Go/No-go tests were included in this procedure as part of a different study, carried out with the same participants, on the role of impulsivity in addiction and motor inhibition (Go/No-go). UPPS-P scores did not exert any effect on reversal learning performance, either by itself or in combination with group (minimum *p* = 0.22). In addition, UPPS-P scores are confounded with addictive behaviors (addictive behaviors are by definition impulsive) so, they were not taken into account for the present study. Still, between-group differences in impulsivity, and the impact of impulsivity on other decision-making tasks have been reported in Torres et al. (2013).

### Instruments

#### Interview for research on addictive behaviors (Spanish version; Verdejo-García et al., 2005)

As noted in the introduction, a key factor in the present study is the degree of dosage-like exposure to cocaine and gambling activities (in the CDI and PG groups, respectively). Psychometric tools developed for clinical purposes do not measure exposure in an isolated manner (disregarding craving intensity, perception of lack of control over the addictive behavior, social and family problems, financial problems, and other symptoms and consequences of addiction).

All of those side factors are irrelevant to the current study. Actually, they would blur drug/gambling exposure effects. Hence, information about lifetime amount and duration of use of the different drugs was collected using the IRAB (Verdejo-García et al., 2005). The IRAB is inspired by applied and experimental behavior analysis, and was not developed to estimate the clinical significance of addiction, but to quantify the most important parameters of drug use behaviors (frequency, duration, amount), independently of the clinical status of the participant and the accompanying symptomatology. All the participants in the three groups went through the full IRAB interview. Here, we will consider the answers to three questions included in the interview: the average frequency of use (times/month), the average amount consumed per episode (in grams or units), and the total duration of the usage period (in months). In accordance with standardized instructions, these parameters were used to compute two composite measures: (1) average monthly amount of each drug consumed (amount × frequency), in grams/month, and (2) severity, or estimated lifetime amount of drug consumed (amount × frequency × duration), in grams or units.

In order to avoid extremely skewed distributions, monthly amount and severity were translated into within-design rank scores for all analyses. A more detailed display of (non-transformed) IRAB results for HC, PG, and CDI can be found in Table A 1 in the Appendix.

Average monthly use is customarily interpreted as an estimate of the *intensity* of addiction during its course (acute exposure). Severity, on the other hand, is attributed the cumulative effect of addiction (chronic exposure). In the case of drugs of abuse, severity is customarily assumed to correlate with the long-term neurotoxic or neuroadaptive effects of that drug (Albein-Urios et al., 2012a).

The IRAB has not been yet developed for gambling activities. Thus, in order to have equivalent measures for gambling and cocaine use, gamblers were asked the same abovementioned IRAB questions (amount, frequency, lifetime duration of usage), but referred to gambling activities. That is, the same questions used in the IRAB for registering drug use, were adapted to estimate the two key gambling parameters (intensity and severity), and then translated into within-design rank scores. In this case, as no toxic substance is involved, severity would correlate with cumulative neuroadaptive or practice-dependent effects of gambling activities (Robinson and Berridge, 2003).

#### The Kaufman brief intelligence test (Kaufman and Kaufman, 1990)

The K-BIT has been standardized and utilized widely, both in clinical and research settings, to assess cognitive abilities. It comprises measures of verbal and non-verbal intelligence and takes 10–30 min to administer. For our purposes, we will use only the compound IQ total score.

#### Probabilistic reversal learning task (Verdejo-García et al., 2010)

The reversal learning task used here is based on the PROB task described in Swainson et al. (2000). A graphical description of the task can be found in Verdejo-García et al. (2010). In each trial of the task, there was a simultaneous presentation of two squares, drawn in different colored lines. The task consisted of four phases in total. In each phase, one stimulus is considered the “correct” one, as choosing it (i.e., mouse-clicking on it) provides reward in most cases, and the other is the “wrong” one, as choosing it was penalized most of the times. This means that, on some trials, the computer provided false feedback, i.e., selecting the correct stimulus was followed by false negative feedback (NF) and selecting the incorrect one was followed by false positive feedback. Positions of stimuli were randomly shifted to avoid motor perseveration. Both negative and positive feedbacks were presented visually, and involved winning or losing five points. The total amount of points accrued was continuously viewed just below the center of the screen. Crucially, the color corresponding to the correct choice and the one corresponding to the wrong choice shifted after every phase (40 trials), that is, the stimulus that was previously correct became incorrect, and vice versa.

For half the participants in each group, the percentage of rewarded clicks on the good option was 75% in Phases 1 and 2, and 87.5% in Phases 3 and 4 (the task became slightly easier in its second half). The other way round, for the other half of participants, the percentage of rewarded clicks on the good option was 87.5% in Phases 1 and 2, and 75% in Phases 3 and 4 (the task became slightly more difficult in its second half). In other words, the order of contingencies was a balanced factor.

### Statistical analysis of PRLT performance

The main dependent variable for global PRLT performance analysis was the number of correct choices (clicks on the highly rewarded option) per each 10-trial block within each 40-trial phase. In a first, full-task analysis, correct choices per phase and block were submitted to a mixed three-factor ANOVA, with phase (1–4) and block (1–4) as within-group factors, and group (HC, PG, CDI) as between-group factor.

Secondly, theory-driven analyses will focus on the number of correct choices in the first block of each phase, and the number of correct choices in the last two blocks of each phase (collapsed). It is important to note that (1) only the number of correct choices in the first block of phases 2–4 can be interpreted as an index of reversal cost. However, block 1 from the first phase will be also included in analyses for design completeness reasons (the inclusion or exclusion of that block does not significantly influence the results of those analyses, nor the main conclusions drawn from them). And (2) the number of correct choices in the two last blocks of each phase can be interpreted as an estimate of asymptotic learning level.^1^

This second series of analysis will be restricted to the impact of chronic and acute exposure to gambling/cocaine in the clinical groups. Four ANCOVAs (with intensity and severity as covariates) were separately carried out for the PG and the CDI groups, with the phase-wise first and last (two) blocks’ correct choices as separate dependent measures.

Similarly to correct choices, decision latencies (measured as reaction times from presentation of the two choice options to the decision made by the participant, averaged for each block) were submitted, firstly, to a block × phase × group global analyses. Subsequently, separate ANCOVAs for PG and CDI groups, with monthly use and severity as joint continuous predictors, were also carried out. Although decision latencies have not been customarily taken into account in reversal learning tasks, we will include them here as complementary evidence.

Given that groups differ in alcohol and cannabis use, prior to all analysis involving the group factor (HC, PG. CDI) we carried out an ANCOVA disregarding the factor group, but including alcohol and cannabis monthly use (translated into rank scores) as continuous covariate predictors, and the same dependent measure used in the corresponding between-group analysis. These pre-analyses were thus carried out for the phase- and block-wise number of correct choices, decision latencies, and FRN magnitudes. As shown in ANCOVAs for the potential effects of cannabis and alcohol use on relevant dependent measures in Appendix, none of the potential confounders (alcohol use, cannabis use, and their interaction) had a significant impact on the abovementioned measures.

For all tests, the significance level was set at 0.05, after Greenhouse–Geisser correction of degrees of freedom where it was necessary.

### EEG recording

EEGs were recorded from 62 scalp locations using tin electrodes arranged according to the extended 10–20 system mounted on an elastic cap (Brain Products, Inc), and referenced online to FCz. Vertical and horizontal eye activity were recorded from one monopolar electrode placed below the left eye, and one monopolar electrode located in a straight line at the outer canthi of the right eye. Two scalp electrodes were attached to mastoids. All electrode impedances during recording were below 5 kΩ. EEG and EOG were sampled at 1000 Hz and amplified using a 0.016–1000 Hz band-pass filter. Subsequently, all EEG recordings were downsampled to 250 Hz, band-pass filtered using a 0.1–25 Hz 12 dB/octave, re-referenced offline to average activity of the mastoids electrodes, and FCz activity was recovered. Offline signal preprocessing was done using EEGLAB software (Delorme and Makeig, 2004) freely available at http://sccn.ucsd.edu/eeglab.

### ERP extraction and analysis

EEG recordings were segmented from −200 to +350 ms, time-locked to the feedback onset. Epochs were corrected for ocular artifacts by computing the SOBI ICA decomposition (Belouchrani et al., 1993, 1997; Cardoso and Souloumiac, 1996, see also Tang et al., 2004), as identified by the ADJUST algorithm (Mognon et al., 2011). Other artifacts were subsequently removed using an automatic rejection procedure: segments were excluded for the remaining analyses when amplitudes were outside the ±100 μV range. Afterward, segments were categorized as belonging to positive- or negative-feedback trials (PF, NF). After the artifact correction procedure, a minimum of 27 trials for the NF and 51 for PF segments were retained for further processing.

Next, the FRN was computed for each participant and feedback condition, as the difference between the average amplitude in the 220–350 ms post-feedback interval, and the preceding positive peak in the 150–220 ms interval. The magnitude of that difference is normally larger for negative than for positive feedback (Hajcak et al., 2005a,b), so a differential FRN score (henceforth, simply FRN score) was computed as the difference between the FRN for PF and the FRN for NF.

Statistical analyses were carried out on FRN scores for Fz and FCz electrodes. The Pz channel was also included to test whether observed effects could be attributed to P3 (as it has been observed that P3 amplitude can affect FRN, and that it is affected by contingency changes; Barcelo et al., 2006). P3 was thus extracted from Fz, FCz, and Pz. However, the time window in which P3 is normally observed includes, in our task, activity evoked by the following trial in the sequence. In order to avoid signal contamination, we carried out P3 analyses on a score computed as the average amplitude for the last 50 ms of each segment referred to the average amplitude during the immediately preceding 100 ms time window (see Chase et al., 2011, for a similar procedure). As we did with the FRN, a differential P3 score (henceforth simply P3 score) was computed as the difference between the P3 scores for NF and PF.

Feedback-related negativity scores were submitted to a 3 (group: CDI, PG, and HC) × 2 (channel: Fz, FCz) repeated-measures analysis of variance. P3 scores were submitted to a 3 (group) × 3 (channel: Fz, FCz, Pz) repeated-measures ANOVA. The Bonferroni procedure was used to correct for multiple comparisons. A 0.05 *p*-level was used for all the statistical decisions. Two participants from the PG group and two from the HC group were excluded from the analysis due to equipment malfunctioning.

### Brain localization

Standardized low-resolution electromagnetic tomography (sLORETA) was used for estimating the 3D cortical distribution of current density underlying scalp activity. sLORETA, computations were done using the MNI152 template, with the 3D space solution restricted to cortical gray matter, according to the probabilistic Talairach atlas. The cortical gray matter is partitioned in 6239 voxels at 5 mm spatial resolution. Brodmann anatomical labels are reported using MNI space. Standardized sLORETA current source densities with no regularization method were obtained from 61 channels (after recovering FCz) for each participant in each condition, and for each time point in each feedback condition. A discussion on the technical details of sLORETA and, specifically, on the necessary restrictions for a viable solution to the inverse problem can be found in Pascual-Marqui (2002).

The identification of the sources with a differential involvement in the generation of FRN across groups followed the rationale recently described by Catena et al. (2012 see also Silton et al., 2009; Torres et al., 2013). A significant correlation across participants between current source density (i.e., estimated activation) at a certain voxel and the magnitude of FRN observed at FCz can be interpreted as indicative of the involvement of such a voxel in the generation of FRN. In other words, the correlations between voxelwise current densities and FRN magnitudes can be used to identify the brain areas involved in the generation of FRN.

Under such an assumption, brain localization analysis was carried out according to the following steps: first, a representative measure of the activation of each voxel for the FRN interval was computed, by averaging voxel activations across the 220–330 post-feedback time window. Second, we computed the correlation (across participants) between that averaged current density and the magnitude of the FRN effect, for each voxel. And third, those areas in which at least 10 voxels were found to significantly correlate with the FRN score were singled out as candidate areas with a functional role in its generation.

## Results

### Behavioral results

#### PRLT: Decision making

**Figure 1:**
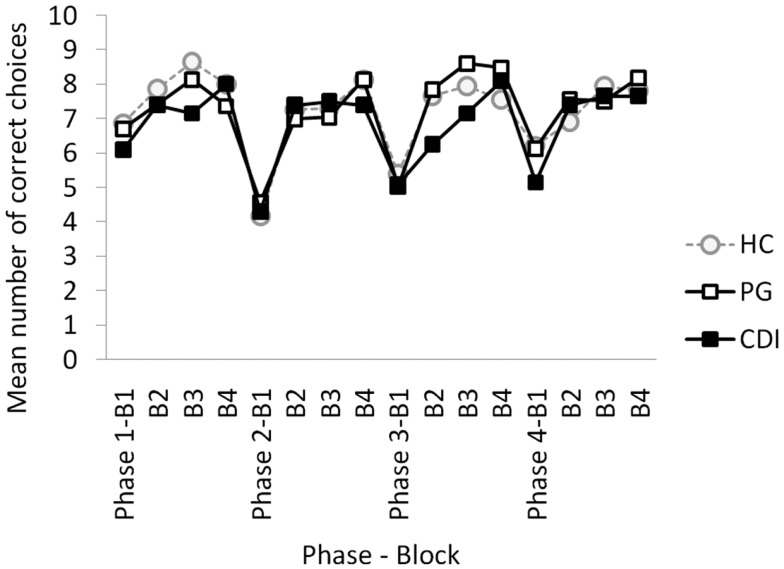
**Mean number of correct choices per block and phase, for HC, healthy controls; PG, pathological gamblers; and CDI, cocaine dependent individuals**.

The main dependent variable in the global analysis was the number of correct choices (clicks on the highly rewarded option) per each 10-trial block and each 40-trial phase. Figure 1 shows the mean number of correct choices per phase, block, and group. The mixed ANOVA, with phase (1–4) and block (1–4) as within-group factors, and group (HC, PG, CDI) as the between-group factor, yielded a significant block × phase × group interaction, *F*(18, 549) = 1.86, MSE = 2.347, *p* = 0.03, η^2^ = 0.06. As expected, there were also significant effects of phase, *F*(3, 183) = 3.06, MSE = 8.88, *p* = 0.04, η^2^ = 0.05, block, *F*(3, 183) = 85.62, MSE = 3.68, *p* < 0.01, η^2^ = 0.58, and phase × block, *F*(9, 549) = 5.416, MSE = 2.35, *p* < 0.03. η^2^ = 0.08, showing within-phase learning effects, and between-phases reversal costs in all groups.

Despite the significant three-way interaction, differences across groups were not significant for any phase and block of the task according to Bonferroni *post hoc* tests. Applying a non-corrected *post hoc* LSD approach, differences between HC and CDI were observed on the second block of the first phase, *t*(41) = 2.40, *p* = 0.02, and between PG and CDI on blocks 2, *t*(39) = 2.07, *p* = 0.04, and 3, *t*(39) = 2.12, *p* = 0.04, of Phase 3.

Results were clearer after taking monthly use and severity scores into account. Given that monthly use and severity refer to different addictive behaviors for PG and CDI, and that HC participants have neither monthly use nor severity scores, we carried out separate repeated-measures ANCOVAs for PG and CDI groups, using severity and monthly amount as covariates, and the number of correct choices in the first block of each phase, and the number of correct choices in the last two blocks of each phase as dependent measures (see Statistical Analysis and footnote 1).

In search of reversal cost effects, we carried out separate ANCOVAs for the two clinical groups, with the number of correct choices in the first block of each phase as dependent measure. In the PG group, the analysis yielded a main effect of monthly amount gambled, *F*(1, 18) = 4.42, MSE = 5.66, *p* = 0.05, η^2^ = 0.19. No other marginal or interactive effects involving monthly amount gambled or gambling severity were close to significance (minimum *p* = 0.16). In the CDI group however, an identical analysis carried out with cocaine monthly use and cocaine use severity as covariates, did not yield any main or interactive significant effect (minimum *p* = 0.44).

Similarly, two ANCOVAs were carried out with asymptotic learning scores as the dependent measure. In the PG group, the analysis did not yield any marginal or interactive significant effect (minimum *p* = 0.18). In the CDI group, on the contrary, the analysis yielded now a significant main effect of cocaine severity, *F*(1, 17) = 4.71, MSE = 4.71, *p* = 0.04, η^2^ = 0.22.

Table 2 shows where the effects yielded by these ANCOVAs originate. The table displays partial correlations – in the PG group – between monthly amount gambled and the number of correct choices in block 1 (phases 1–4), with gambling severity as variable of control; and – in the CDI group – between cocaine use severity and the asymptotic learning measure, with cocaine monthly use as control variable. The effect of monthly amount gambled on first block correct choices was actually restricted to phases 2 and 4, namely, to the first and the third reversals of the task. Cocaine use severity, in turn, exerted its effect on phases 3 and 4.

**Table 2:** **Partial correlations between monthly amount gambled and number of correct choices in block 1 (phases 1–4), and between cocaine use severity and number of correct choices in blocks 3/4 (phases 1–4).)**.

	Phase 1		Phase 2		Phase 3		Phase 4	
	partial *r*	α	partial *r*	α	partial *r*	α	partial *r*	α
Gambling MU – correct choices (block 1)	−0.12	0.63	−**0.50**	0.03*	−0.17	0.49	−**0.46**	0.05*
Cocaine severity – correct choices (blocks 3/4)	−0.31	0.21	−0.24	0.33	−**0.69**	0.01*	−**0.51**	0.03*

*Correlations larger than 0.46 (in absolute terms are bilaterally significant. Values in bold stand for statistically significant correlations and their corresponding p-values*.

### Decision latencies

Finally, we analyzed the effects of group, monthly use, and severity on decision latencies. The main dependent measure was the mean decision latency per phase and block. The group (HC, PG, CDI) × phase (1–4) × block (1–4) ANOVA did not show any significant marginal or interactive effect of group (minimum *p* = 0.328).

The ANCOVA for the PG group, with block and phase as within-group variables, and monthly amount gambled and severity scores as continuous predictors, showed significant main effects of the monthly amount gambled, *F*(1, 18) = 5.66, *p* = 0.03, η^2^ = 0.24 and gambling severity, *F*(1, 18) = 4.81, *p* = 0.04, η^2^ = 0.21. No other marginal or interactive effect was close to significance (minimum *p* = 0.24). An analogous ANCOVA on CDI decision latencies, and cocaine monthly use and cocaine severity as covariates, did not show any significant effect of monthly use, or severity (all *p* > 0.10).

**Figure 2:**
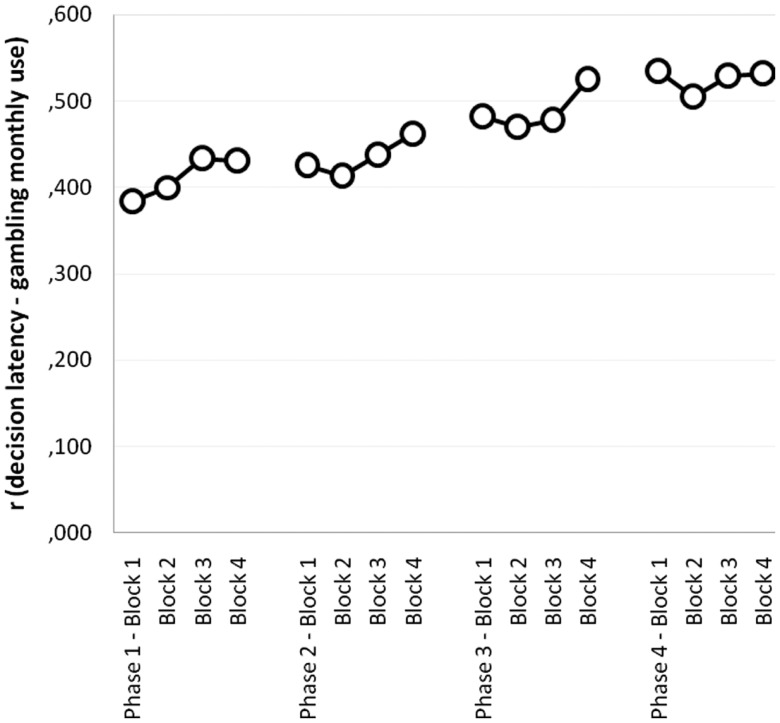
**Partial correlations between monthly amount gambled and decision latency per phase and block (controlling for gambling severity), in the PG group**.

Figure 2 shows a graphical depiction of the monthly amount effect observed in the PG group (coefficients represent partial correlations between monthly amount gambled and decision latency for each phase and block, computed while controlling for severity). Consistently across the task, the monthly amount gambled positively covaried with decision latency, which means that the intensity of addiction slowed decisions down (all correlations above 0.445 are bilaterally significant).

In summary, the clinical groups seem to show different learning dynamics in the reversal learning task when compared to matched controls. However, such differences cannot be fully characterized if addictions are not considered from an idiosyncratic point of view (i.e., taking chronic and acute exposure into account).

Gambling intensity, measured as the monthly amount gambled, emerges as a powerful mediator of learning-driven decision-making: heavier gamblers tend to show signs of enhanced reversal cost, and, additionally, tend to make significantly slower predictions. Increased latency in decision-making tasks is customarily interpreted as a sign of decisional difficulty (Spinoza-Varas and Watson, 1994), although this measure has been paid no attention at all in reversal learning studies.

The severity of gambling did not exert any significant effect on reversal cost, which implies that the effect of gambling on that particular aspect of reversal learning is not cumulative. On the other hand, cocaine use severity, but not intensity, interfered with asymptotic-level decision making. In this case, the potential effects on decision latencies were negligible.

### EEG results

#### Feedback-related negativity

**Figure 3:**
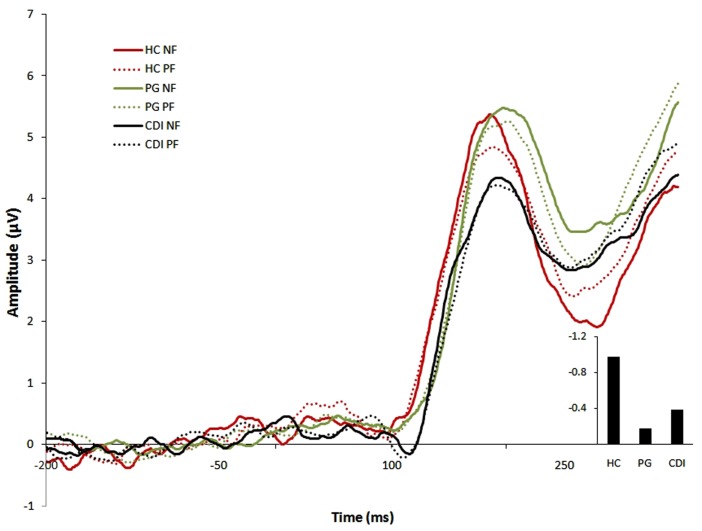
**ERP waveforms for each group in each feedback condition**. Right bottom panel: differential FRN effect for the three groups. All amplitudes are in μV. HC, Healthy controls; PG, Pathological gamblers; CDI, Cocaine dependent individuals; NF, Negative feedback; PF, Positive feedback.

Figure 3 displays ERP waveforms for each group in each feedback condition. The 3 (group: HC, PG, CDI) × 2 (channel: Fz, FCz) mixed ANOVA on the FRN score yielded significant main effects of group, *F*(2, 57) = 4.04, MSE = 1.39, *p* < 0.03, η*^2^* = 0.12, and Channel, *F*(1, 57) = 20.19, MSE = 0.45, *p* < 0.01, η*^2^* = 0.26, being the largest FRN score observed at FCz. There was no interaction between the two factors, *F*(2, 57) = 0.51. Bonferroni-corrected *post hoc* comparisons showed that the FRN score was larger for HC than for PG (*p* = 0.02). No other effects were significant. With regard to P3, there was a theoretically irrelevant effect of channel, *F*(2, 116) = 3.19, MSE = 1.17, *p* < 0.05, η*^2^* = 0.05, but both the group effect, *F*(2, 58) = 0.92, and the group × channel interaction, *F*(4, 58) = 0.97, were very far from the significance level.

#### Source location

Using the bootstrapping approach (included in the sLoreta package) we observed several right hemisphere clusters of voxels that significantly correlated with FRN scores in the control group (Table 3): the inferior (BA46) and middle (BA9 and BA10) frontal gyri, the insula (BA13), and the posterior cingulate gyrus (BA23). As noted in the Section “Materials and Methods,” we take this as evidence of the involvement of these areas in the generation of FRN in normal conditions (please note that negative correlations imply that the larger the activation in these areas, the larger – in absolute values – the FRN score). These areas were established as regions of interest to detect differences between the clinical groups.

**Table 3:** **Brain areas significantly correlated to the FRN score in the control group**.

Lobe	Structure	BA	*k*	*X*	*Y*	*Z*	CSD-FRN Correlation
Frontal	Middle frontal	9	10	20	35	20	−0.70
Frontal	Middle frontal	10	16	35	40	15	−0.75
Sub-lobar	Insula	13	9	40	15	15	−0.71
Limbic	Posterior cingulate	23	4	5	−40	25	−0.75
Frontal	Inferior frontal gyrus	46	16	35	35	15	−0.74

*BA, Brodmann area; k, cluster size in voxels; *X*, *Y*, and *Z* are in MNI space*.

Feedback-related negativity-current density correlations in those same areas for the two clinical groups are reported in Table 4. Not surprisingly, those correlations differed from the ones in the control group (*p* = 0.14, 0.06, 0.19, 0.06, 0.04 for the HC versus CDI contrasts; and *p* < 0.01 for all HC versus PG contrasts across areas). The deviation was thus larger for PG than for CDI. Density-FRN correlations in the CDI group, although lower, were in the same direction than the ones observed in the HC group. Correlations in the PG group were mostly in the opposite direction, and (according to the Bonferroni correction) significantly differed from CDI’s in BA9, BA10, BA13, and BA23 (Table 4, rightmost column).

**Table 4:** **FRN-current density correlation coefficients for the key areas involved in FRN generation (as detected in controls), and significance of Bonferroni-corrected contrasts between correlation coefficients across groups (PG, Pathological gamblers; CDI, Cocaine dependent individuals)**.

*Structure*	BA	CDI	PG	*p* (PG versus CDI)
Middle frontal	9	−0.47	0.35	<0.01*
Middle frontal	10	−0.43	0.34	0.01*
Insula	13	−0.53	0.72	<0.01*
Posterior cingulate	23	−0.42	0.78	<0.01*
Inferior frontal gyrus	46	−0.36	0.07	0.10°

°Non-significant; *p < 0.05. p values in bold correspond to significant differences between PGs and CDIs.

## Discussion

Our first research aim was to check for the existence of anomalies in reversal learning in two groups of CDI and PG, when compared against HC. Such anomalies have been only partially corroborated. The group × block × phase interaction effect on correct decisions indicates that learning progressed differently in the three groups. Such a difference is, however, subtle. In specific points of the task, individuals in the CDI group performed worse than controls (phase 1, block 2), or than PG participants (phase 3, blocks 2 and 3). The observation that cocaine addicts are globally (although only slightly) more hampered than other groups in PRLT performance is fully coincident with the results reported by Fernández-Serrano et al. (2012). Additionally, the significance of such a difference is strengthened by the existence of differences at the electroencephalographic level, as discussed later.

In relation to our second research aim, group analyses demonstrate that the difficulty to interpret between-group PRLT performance differences can be due – at least in part – to differences within the clinical groups. On the one hand, asymptotic learning, as measured by the averaged number of correct choices in the two last blocks of each phase, was significantly affected by cocaine severity, that is, by the estimated cumulative exposure to cocaine during the course of the addictive process.

On the other hand, reversal costs (as observed in phases 2 and 4; see Table 2) were specifically associated to gambling intensity, namely, to the averaged amount gambled per unit of time. Those gamblers who spend more money in gambling activities also tend to show larger reversal costs. This is compatible with Leeman and Potenza’s (2012) proposal that there is a privileged link between gambling and learning inflexibility^2^. Additionally, we provide evidence that gambling, but not cocaine use, slows decisions down. Increased latency in decision-making tasks is customarily interpreted as a sign of decision difficulty (Spinoza-Varas and Watson, 1994). So, this finding supports the idea that gambling is specifically linked to the decisional aspects of reversal learning. This association between gambling intensity, reversal cost, and increased decision difficulty probably deserves further research.

The fact that the monthly amount gambled (i.e., gambling intensity), but not gambling severity, exerts a significant impact on phase-by-phase first block correct choices implies that the gambling effect on such measure is not cumulative, that is, not due to practice with gambling scenarios, or chronic gambling-induced neuroadaptation. In other words, it is unlikely that increased reversal costs are attributable to practice or sensitization. Conversely, the evidence that cocaine use severity, but not monthly use (i.e., intensity), exerts an impact on asymptotic reversal learning seems to prove that the cumulative effect of cocaine exposure (neurotoxicity) is exerted on a different component of reversal learning performance, not necessarily involving learning inflexibility. Relatedly, Albein-Urios et al. (2012a) and Torres et al. (2013) have recently demonstrated that some other well-known neuropsychological anomalies observed in CDI (e.g., working memory and motor inhibition deficits) are also attributable to cocaine neurotoxic effects.

Still, the interpretation of our PRLT behavioral results requires some further considerations. Firstly, recent evidence (van Holst et al., 2010; Shaffer and Martin, 2011) shows that there exist non-trivial psychological differences underlying differential preferences for low-rate high-stakes gambling modalities (casino games, sport bets), versus high-rate low-stakes ones (Video-lottery terminals, slot machines). Our sample mostly consisted of male slot machine gamblers, and was not large enough to segregate these two categories. At this moment, the role of gambling preferences in PRLT performance remains open. And secondly, in most implementations of the PRLT reversals do not occur at fixed times, but when the participant have reached a pre-established learning criterion (for example, five-correct choices in a row; Franken et al., 2008). Performance is then assessed as the total number of reversals during the task, the total number of incorrect choices, the mean number of trials-to-criterion, or the mean number of incorrect choices in a row after a reversal (perseverative series; Franken et al., 2008; de Ruiter et al., 2009; Camchong et al., 2011; Lucantonio et al., 2012). These measures are customarily interpreted as measures of reversal cost or reversal learning (in)flexibility.

In our version of the task, phase length was fixed (40 trials), to ensure comparability of learning curves across groups in global analyses (and consequently to make the distinction between reversal cost and asymptotic learning possible). In addition, our main flexibility-related dependent measure was the number of correct choices in phase-by-phase first blocks. The reason underlying the use of such measure (instead of the more common perseverative series mean length), is strictly statistical: given that the PRLT provides probabilistic false feedback (punishment for a correct choice) the length of error series tends to be very variable within each participant, depending on the particular ordering of trials in the series. Most PRLT implementations do not warrant asymptotic learning, but allow for a high number of reversals, so variability can be reduced by means of averaging. In our case, the task ensures asymptotic learning in each phase (see footnote 1), but contains only three contingency reversal points, and thus a more stable measure is required. This particularity, however, does not compromise the interpretation of the measure in terms of reversal cost/learning inflexibility (at least for blocks 2–4).

Our third and last research aim was to analyze electroencephalographic differences between-groups (and, particularly, the differences between the two clinical groups) with regard to their response to feedback during reversal learning. We have observed abnormal feedback-evoked cortical activity in the PG group. If we take the magnitude and sign of the differential FRN score in the control group as a reference of normality, the deviation from that reference was maximal for gamblers (the FRN was visually smaller for CDI than for HC, but CDI did not statistically differ either from HC or from the PG).

According to Hajcak et al. (2005a,b), the FRN is mainly elicited by unexpected negative outcomes (see also Holroyd et al., 2004), and reflects the binary evaluation of good versus bad outcomes. If that interpretation is taken as correct, it implies that gamblers are particularly hampered to adequately ponder the impact of NF. Consequently, we can assume that they are also hampered to learn to make decisions on the individual history of losses. This is fully coincident with the finding that gambling slows decisions down, and also with our separate finding that recreational gamblers, at non-pathological levels, are less sensitive to losses than non-gamblers (Torres et al., submitted).

Results regarding source location point in the same direction. In accordance with the results we have obtained with HC, Hampshire et al. (2012) found several areas to be particularly active when reversal events were compared against other switch events (i.e., changes in the set of stimuli). These areas included the most posterior extent of the right inferior frontal gyrus, extending into the anterior insula, and the frontopolar portion of the middle frontal gyrus (see also Mitchell et al., 2008), and were more active when NF led to a change in the response pattern. In that study, the dorsolateral prefrontal cortex was also found to be involved in reversal events, whereas other studies have attributed to it more general higher-order executive functions involving attention (Reminjse et al., 2005), and coordination of search behavior (Hampshire and Owen, 2006). In any case, this set of anatomical areas is almost fully coincident with the ones found to be involved in the generation of the FRN in HC in the present study.

The only discordance between the present and previous results seems to be the involvement of the posterior portion of the cingulate gyrus in the generation of FRN. D’Cruz et al. (2011), and Robinson et al. (2010) found the activation of posterior cingulate cortex after positive feedback in the reversal learning task to depend on whether it was expected or not. In a work by Gläscher et al. (2009), activation in the same area was associated to the experienced value of the chosen option. Relatedly, Nashiro et al. (2012) found it to be more active when feedback was emotional than when it was neutral. And finally, a study by Albein-Urios et al. (2012b), found it to be involved in regulation of negative emotions. So, it is plausible that variability in the magnitude of FRN is associated to emotional aspects of feedback valuation.

Most importantly when these areas were taken as regions of interest for the clinical groups, PG strikingly differed from CDI. Although, as noted above, the magnitude of FRN did not differ between the clinical groups, sLORETA analyses unveiled differences in the involvement of these areas in FRN generation. There is ample evidence that addictive processes are associated to abnormal response to feedback and abnormal activation of prefrontal and orbitofrontal areas (see Schoenbaum et al., 2006, for a review). However, our results provide the first direct neuroanatomical evidence in favor of Leeman and Potenza’s (2012) proposal that reversal learning deficits are particularly severe in gamblers (when compared against other populations of addicted individuals).

Despite its several specific strengths (the careful selection of participants, the close matching between-groups in intellectual functioning and sociodemographic variables and the absence of comorbidities in them), this study has also some limitations that are worth mentioning. One of them is undoubtedly the absence of enough trials in the reversal learning task to track changes in the FRN across the task, and, more specifically, to clearly separate between reversal errors (those occurring in the first trials after reversal points) and errors spontaneously occurring during other parts of the task. We have shown that learning dynamics are behaviorally relevant; further research is needed to describe in a similarly detailed way the evolving changes in cortical activity occurring in parallel with such learning dynamics. A second limitation is the impossibility to separate gamblers with preferences for different games of chance. Third, despite the careful selection of participants, it is virtually impossible to match groups in every potentially relevant factor. Specifically, potentially addictive behaviors tend to show complex correlation patterns. In our case, cocaine users were also more likely to use alcohol and cannabis than gamblers and controls. Although cannabis and alcohol use did not exert any direct effect on PRLT performance or cortical activity, the possibility exists that these drugs modulated the chronic effects of gambling/cocaine. This limitation is common to virtually all studies in which the group comparison methodology is used. And finally, despite the recording of drug/gambling exposure measures, group comparison studies are less conclusive than prospective studies, with regard to the possibility to establish directional causal links between neuropsychological abnormalities and addictive behaviors. These four potential weaknesses warrant further research.

## Final Remarks

To date, results regarding reversal learning deficits in addicts have been elusive. This work confirms previous proposals that feedback-based instrumental learning is more inflexible in pathological gambling than in other forms of addiction, such as cocaine dependence. At the same time, however, it raises important questions about the causes of such inflexibility and the role within-group variability in clinical samples. At the behavioral level, the main findings of this research point out that gambling severity slows decisions and increases reversal cost, whereas cocaine addiction affects asymptotic scores in reversal learning tasks. More importantly, psychophysiological and neuroanatomical data provided the first direct evidence that reversal learning deficits in gamblers differ from drug addicts’ and are related to abnormal activity in specific prefrontal and orbitofrontal areas.

## Conflict of Interest Statement

The authors declare that the research was conducted in the absence of any commercial or financial relationships that could be construed as a potential conflict of interest.

## Footnotes

^1^The use of the averaged two last blocks as an index of asymptotic learning rests on the assumption that no further learning occurs in any of the three groups after block 3 (so learning can be considered maximal in blocks 3 and 4). Actually, taking into account those two blocks only, there was neither effect of block [F(1, 61) = 2.49, MSE = 1.73, p = 0.12] nor block × group interaction [F(1, 61) = 1.65, MSE = 1.73, p =0.20]. Mean (SE) number of correct choices were 8.02 (0.26), 7.80 (0.26), 7.28 (0.32), 7.91 (0.27), 7.92 (0.26), 8.03 (0.23), 7.72 (0.23), and 7.89 (0.25) for phase 1 block 3, phase 1 block 4, phase 2 block 3, phase 2 block 4, phase 3 block 3, phase 3 block 4, phase 4 block 3, an phase 4 block 4, respectively.

^2^On the contrary, Ersche et al. (2008) made a very careful evaluation of a number of performance indices and found true reversal cost in cocaine users (but not in amphetamine users). Importantly, that effect was found in *current* cocaine users, but not in abstinent ex-addicts. At difference with gambling, the existence of reversal costs associated to chronic effects of cocaine addiction remains undemonstrated.

## Appendix

The IRAB provides information on frequency of drug use, amount per drug use episode, and duration of use. The combination of frequency and amount per episode allows for an estimate of the amount used per unit of time. Duration of use is customarily expressed in months, whereas amount units vary across substances. Severity is computed as the monthly use × duration product (note that severity scores can reach extremely high values, so correction measures are taken for analysis; e.g., translating them into ranks, computing them from standardized duration and monthly use scores).

Table A1 displays the observed IRAB results for regular use of cannabis, tobacco, alcohol, and cocaine, as well as the equivalent measures of gambling. Only those drugs of abuse used once a month by at least 15% of the individuals in any of the samples have been included. Mean consumption of all other drugs included in the IRAB (MDMA, amphetamine, methamphetamine, heroine, benzodiazepines, hallucinogens) is negligible. The number of illegal drugs used at least once in the whole lifetime, however, provides extra information about the different drug use patterns in the three groups.

**Table A1:** **IRAB Results for healthy controls, pathological gamblers, and cocaine dependent individuals**.

	Cannabis		Cocaine		Tobacco		Alcohol			Gambling	
	Monthly use (joints)	Duration (months)	Monthly use (grams)	Duration (months)	Monthly use (cigarettes)	Duration (months)	Monthly use (units)	Duration (months)	Number of illegal drugs used (lifetime)	Monthly use (euros)	Duration (months)
HC	13.00	39.83	0.33	4.96	225.04	51.26	44.02	126.26	2.17	–	–
	(26.61)	(78.14)	(1.01)	(14.28)	(266.50)	(73.80)	(42.81)	(91.52)	(1.97)	–	–
PG	8.39	24.06	0.45	16.72	373.06	112.56	99.14	152.94	1.56	1503.67	93.43
	(28.15)	(52.68)	(1.35)	(49.05)	(362.37)	(95.80)	(80.12)	(67.11)	(1.72)	(693.32)	(66.18)
CDI	67.00	91.95	18.69	102.90	339.40	156.00	158.20	156.95	4.60	6.45	13.80
	(63.71)	(81.42)	(11.99)	(75.02)	(291.41)	(92.95)	(118.88)	(91.51)	(1.67)	(20.09)	(53.85)

### ANCOVAs for the potential effects of cannabis and alcohol use on relevant dependent measures

Either monthly use of cannabis and alcohol, or severity of alcohol/cannabis consumption could have been used as covariates for the analyses reported in this appendix. However, severity and monthly use scores (once translated into ranks) were virtually co-linear (severity-monthly use correlation was *r* = 0.754 for alcohol, and *r* = 0.937 for cannabis). The joint inclusion of severity and monthly use measures in the following ANCOVAs would imply a violation of this analysis’ criteria, and would thus lead to inconsistent results.

#### PRLT: Decision-making

The within-subject pre-analysis ANCOVA, with block and phase as within-group factors, and alcohol and cannabis monthly use (translated into rank scores) as covariates did not yield any direct or interactive significant effects for any of the covariates. Only the block × phase × alcohol monthly use approached significance (*p* = 0.10; all other *F* < = 1).

#### PRLT: Decision latencies

As in the previous case, the ANCOVA pre-analysis with phase and block as within-group variables, cannabis and alcohol monthly use as continuous predictors, and phase- and block-wise decision latencies as dependent measure, did not yield any main or interactive effect of the covariates (minimum *p* = 0.186).

#### FRN Scores

As we did with behavioral measures, we carried out a pre-analysis ANCOVA on FRN scores, with channel as the within-group factor, and alcohol and cannabis monthly use (translated into rank scores) as covariates. This analysis did not yield any significant main or interactive effects of alcohol or cannabis (all *p* > 0.38)^.^ Regarding P3 scores, only a theoretically irrelevant alcohol monthly use × channel interaction was observed, *F*(2, 112) = 4.39, *p* = 0.02. Again, alcohol and cannabis use can be discarded as potential causes of group effects.
